# (*S*)-2-(1*H*-Imidazol-1-yl)succinic acid

**DOI:** 10.1107/S1600536809015220

**Published:** 2009-04-30

**Authors:** Jing-Mei Xiao

**Affiliations:** a Ordered Matter Science Research Center, College of Chemistry and Chemical Engineering, Southeast University, Nanjing 211189, People’s Republic of China

## Abstract

The title compound, C_7_H_8_N_2_O_4_, is a zwitterion, [formal name = (*S*)-3-carb­oxy-2-(imidazol-3-ium-1-yl)propano­ate], in which the deproton­ated negatively charged carboxyl­ate end shows almost identical C—O bond distances [1.248 (4) and 1.251 (4) Å] due to resonance. The mol­ecules are involved in inter­molecular O—H⋯O and N—H⋯O hydrogen bonds, which define a tightly bound three-dimensional structure.

## Related literature

For the use of imidazol-1-ylalkanoic acids as probes to determine the intra­cellular and extracellular pH and cell volume by ^1^H NMR, see: López *et al.*(1996 ▶). For the preparation of the title compound, see: Bao *et al.* (2003 ▶).
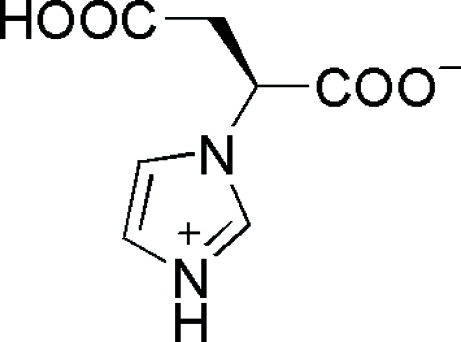


## Experimental

### 

#### Crystal data

C_7_H_8_N_2_O_4_
*M* *_r_* = 184.15
Orthorhombic, 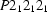

*a* = 7.3212 (16) Å
*b* = 7.9193 (16) Å
*c* = 14.254 (3) Å
*V* = 826.4 (3) Å^3^
*Z* = 4
Mo *K*α radiation
μ = 0.12 mm^−1^
*T* = 293 K
0.25 × 0.20 × 0.18 mm

#### Data collection

Rigaku Mercury2 diffractometer
Absorption correction: multi-scan (*CrystalClear*; Rigaku, 2005 ▶) *T*_min_ = 0.97, *T*_max_ = 0.98
8489 measured reflections
1110 independent reflections
952 reflections with *I* > 2σ(*I*)
*R*_int_ = 0.053

#### Refinement

*R*[*F* ^2^ > 2σ(*F* ^2^)] = 0.050
*wR*(*F* ^2^) = 0.151
*S* = 1.12
1110 reflections
118 parameters
H-atom parameters constrained
Δρ_max_ = 0.19 e Å^−3^
Δρ_min_ = −0.23 e Å^−3^

### 

Data collection: *CrystalClear* (Rigaku, 2005 ▶); cell refinement: *CrystalClear*; data reduction: *CrystalClear*; program(s) used to solve structure: *SHELXS97* (Sheldrick, 2008 ▶); program(s) used to refine structure: *SHELXL97* (Sheldrick, 2008 ▶); molecular graphics: *SHELXTL* (Sheldrick, 2008 ▶); software used to prepare material for publication: *SHELXL97*.

## Supplementary Material

Crystal structure: contains datablocks I, global. DOI: 10.1107/S1600536809015220/bg2234sup1.cif

Structure factors: contains datablocks I. DOI: 10.1107/S1600536809015220/bg2234Isup2.hkl

Additional supplementary materials:  crystallographic information; 3D view; checkCIF report

## Figures and Tables

**Table 1:** Hydrogen-bond geometry (Å, °)

*D*—H⋯*A*	*D*—H	H⋯*A*	*D*⋯*A*	*D*—H⋯*A*
N2—H2⋯O2^i^	0.86	1.91	2.716 (4)	155
O3—H3*C*⋯O1^ii^	0.86	1.71	2.572 (3)	177

Symmetry codes: (i) 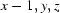; (ii) 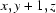.

**Fig. 1.**
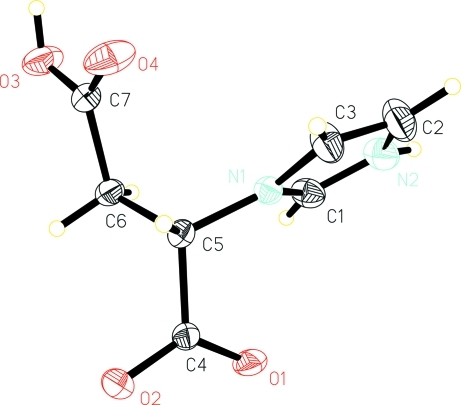
The molecular structure of the title compound, showing the atomic numbering scheme. Displacement ellipsoids are drawn at the 30% probability level.

## supplementary crystallographic information

### Comment

Imidazol-1-ylalkanoic acids are used as new probes  to determine the intracellular and extracellular pH and cell volume by ^1^H NMR. (López *et al.*, 1996). In this report we present the structure of (*S*)-2-(1*H*-imidazol-1-yl)succinic acid. As shown in Fig. 1, the title compound C_7_H_8_N_2_O_4_ exists in the form of an inner salt where the unprotonated, negatively charged carboxylato end shows almost identical C-O bond distances (1.248 (4) and 1.251 (4)Å respectively) due to resonance.. The molecules are involved in intermolecular O—H···O and N—H···O hydrogen bonds (Table 1)  which define a tightly bound 3D structure.

### Experimental

The ligand was prepared according to a literature method (Bao *et al.*, 2003). A formaldehyde water solution (36%, 1.67 g) and a glyoxal water solution (32%, 3.62 g) were mixed in a 50 ml, three-necked flask provided with a stirrer and a reflux condenser. While the mixture was heated at 50 °C with stirring, a mixture of *L*-2-aminosuccinic acid (2.66 g, 0.02 mol), ammonia solution (28%, 1.21 g) and sodium hydroxide solution (10%, 8 g) was added in small portions during 0.5 h. After the mixture was stirred for an additional 8 h at 50 °C, the cooled mixture was acidified to pH=3 with concentrated hydrochloric acid. After stirring for 30 min, the suspension was filtered. The resulting solid was washed with H_2_O and dried in vacuum over P2O5 at room temperature. Colourless crystals suitable for X-ray diffraction were obtained from a solution of 100 mg in 15 ml H_2_O by slow evaporation after one month.

### Refinement

Positional parameters of all the H atoms except for H3C were calculated geometrically and the H atoms were set to ride on the C and N atoms to which they are bonded, with Uiso(H) = 1.2Ueq(C or N). The carboxyl H3C was initially refined and subsequently allowed to ride with Uiso(H) = 1.5Ueq(O). Due to the abscence of anomalous diffraction effects, Friedel pairs were merged.

### Crystal data

**Table:**

C_7_H_8_N_2_O_4_	*F*(000) = 384
*M**_r_* = 184.15	*D*_x_ = 1.480 Mg m^−^^3^
Orthorhombic, *P*2_1_2_1_2_1_	Mo *K*α radiation, λ = 0.71073 Å
Hall symbol: p 2ac 2ab	Cell parameters from 2123 reflections
*a* = 7.3212 (16) Å	θ = 2.8–27.4°
*b* = 7.9193 (16) Å	µ = 0.12 mm^−^^1^
*c* = 14.254 (3) Å	*T* = 293 K
*V* = 826.4 (3) Å^3^	Prism, colorless
*Z* = 4	0.25 × 0.20 × 0.18 mm

### Data collection

**Table:**

Rigaku Mercury2 diffractometer	1110 independent reflections
Radiation source: fine-focus sealed tube	952 reflections with *I* > 2σ(*I*)
graphite	*R*_int_ = 0.053
CCD_Profile_fitting scans	θ_max_ = 27.5°, θ_min_ = 2.9°
Absorption correction: multi-scan (*CrystalClear*; Rigaku, 2005)	*h* = −9→9
*T*_min_ = 0.97, *T*_max_ = 0.98	*k* = −10→10
8489 measured reflections	*l* = −18→18

### Refinement

**Table:**

Refinement on *F*^2^	Primary atom site location: structure-invariant direct methods
Least-squares matrix: full	Secondary atom site location: difference Fourier map
*R*[*F*^2^ > 2σ(*F*^2^)] = 0.050	Hydrogen site location: inferred from neighbouring sites
*wR*(*F*^2^) = 0.151	H-atom parameters constrained
*S* = 1.12	*w* = 1/[σ^2^(*F*_o_^2^) + (0.0852*P*)^2^ + 0.2196*P*] where *P* = (*F*_o_^2^ + 2*F*_c_^2^)/3
1110 reflections	(Δ/σ)_max_ < 0.001
118 parameters	Δρ_max_ = 0.19 e Å^−^^3^
0 restraints	Δρ_min_ = −0.23 e Å^−^^3^

### Special details

**Table:**

Geometry. All esds (except the esd in the dihedral angle between two l.s. planes) are estimated using the full covariance matrix. The cell esds are taken into account individually in the estimation of esds in distances, angles and torsion angles; correlations between esds in cell parameters are only used when they are defined by crystal symmetry. An approximate (isotropic) treatment of cell esds is used for estimating esds involving l.s. planes.
Refinement. Refinement of *F*^2^ against ALL reflections. The weighted *R*-factor *wR* and goodness of fit *S* are based on *F*^2^, conventional *R*-factors *R* are based on *F*, with *F* set to zero for negative *F*^2^. The threshold expression of *F*^2^ > σ(*F*^2^) is used only for calculating *R*-factors(gt) *etc*. and is not relevant to the choice of reflections for refinement. *R*-factors based on *F*^2^ are statistically about twice as large as those based on *F*, and *R*- factors based on ALL data will be even larger.

### Fractional atomic coordinates and isotropic or equivalent isotropic displacement parameters (Å^2^)

**Table:**

	*x*	*y*	*z*	*U*_iso_*/*U*_eq_	
C1	0.9679 (4)	0.2336 (4)	0.6099 (2)	0.0299 (7)	
C2	0.9073 (4)	0.4110 (4)	0.6415 (2)	0.0309 (7)	
H2A	0.9846	0.4450	0.6943	0.037*	
C3	0.9360 (5)	0.5376 (4)	0.5624 (2)	0.0367 (7)	
H3A	1.0554	0.5197	0.5346	0.044*	
H3B	0.8447	0.5193	0.5142	0.044*	
C4	0.9226 (5)	0.7177 (4)	0.5977 (2)	0.0378 (7)	
C5	0.6549 (5)	0.4474 (5)	0.7626 (3)	0.0493 (10)	
H5	0.7259	0.4826	0.8130	0.059*	
C6	0.4741 (6)	0.4230 (7)	0.7635 (3)	0.0640 (13)	
H6	0.3962	0.4380	0.8144	0.077*	
C7	0.5708 (5)	0.3653 (5)	0.6236 (3)	0.0422 (8)	
H7	0.5719	0.3333	0.5608	0.051*	
N1	0.7172 (3)	0.4110 (3)	0.67364 (18)	0.0314 (6)	
N2	0.4268 (4)	0.3725 (4)	0.6766 (2)	0.0507 (8)	
H2	0.3175	0.3487	0.6589	0.061*	
O1	0.8474 (4)	0.1251 (3)	0.5944 (2)	0.0507 (7)	
O2	1.1355 (3)	0.2163 (3)	0.59668 (16)	0.0409 (6)	
O3	0.9107 (5)	0.8279 (3)	0.52925 (19)	0.0584 (9)	
H3C	0.8908	0.9290	0.5489	0.070*	
O4	0.9181 (5)	0.7543 (4)	0.67883 (19)	0.0638 (9)	

### Atomic displacement parameters (Å^2^)

**Table:**

	*U*^11^	*U*^22^	*U*^33^	*U*^12^	*U*^13^	*U*^23^
C1	0.0279 (14)	0.0283 (15)	0.0334 (15)	0.0011 (12)	−0.0001 (12)	0.0036 (13)
C2	0.0315 (15)	0.0274 (15)	0.0338 (15)	−0.0038 (13)	0.0014 (13)	−0.0015 (12)
C3	0.0455 (18)	0.0265 (15)	0.0382 (16)	0.0006 (15)	0.0092 (16)	0.0019 (12)
C4	0.0396 (17)	0.0287 (15)	0.0452 (18)	0.0001 (15)	0.0057 (15)	0.0020 (14)
C5	0.0390 (19)	0.064 (3)	0.045 (2)	−0.0105 (19)	0.0083 (16)	−0.0169 (19)
C6	0.055 (2)	0.077 (3)	0.061 (2)	−0.014 (2)	0.024 (2)	−0.022 (3)
C7	0.0345 (16)	0.0433 (19)	0.0486 (18)	0.0050 (17)	−0.0069 (16)	−0.0066 (15)
N1	0.0293 (13)	0.0301 (13)	0.0349 (14)	−0.0001 (11)	−0.0025 (11)	−0.0032 (11)
N2	0.0310 (14)	0.0487 (18)	0.072 (2)	−0.0010 (15)	−0.0041 (16)	−0.0126 (16)
O1	0.0411 (13)	0.0241 (12)	0.087 (2)	−0.0010 (10)	0.0034 (14)	−0.0071 (13)
O2	0.0350 (12)	0.0383 (13)	0.0494 (14)	0.0044 (10)	0.0047 (11)	−0.0035 (11)
O3	0.090 (2)	0.0294 (13)	0.0560 (15)	0.0060 (15)	0.0122 (16)	0.0052 (11)
O4	0.106 (3)	0.0381 (14)	0.0472 (15)	0.0013 (17)	−0.0113 (17)	−0.0097 (12)

### Geometric parameters (Å, °)

**Table:**

C1—O2	1.249 (4)	C5—C6	1.338 (6)
C1—O1	1.251 (4)	C5—N1	1.378 (4)
C1—C2	1.541 (4)	C5—H5	0.9300
C2—N1	1.465 (4)	C6—N2	1.347 (6)
C2—C3	1.522 (4)	C6—H6	0.9300
C2—H2A	0.9800	C7—N2	1.299 (5)
C3—C4	1.516 (4)	C7—N1	1.338 (4)
C3—H3A	0.9700	C7—H7	0.9300
C3—H3B	0.9700	N2—H2	0.8600
C4—O4	1.193 (4)	O3—H3C	0.8601
C4—O3	1.312 (4)		
			
O2—C1—O1	126.3 (3)	O3—C4—C3	112.6 (3)
O2—C1—C2	115.3 (3)	C6—C5—N1	107.9 (4)
O1—C1—C2	118.3 (3)	C6—C5—H5	126.1
N1—C2—C3	111.3 (3)	N1—C5—H5	126.1
N1—C2—C1	111.4 (2)	C5—C6—N2	106.7 (3)
C3—C2—C1	110.1 (2)	C5—C6—H6	126.6
N1—C2—H2A	108.0	N2—C6—H6	126.6
C3—C2—H2A	108.0	N2—C7—N1	109.1 (3)
C1—C2—H2A	108.0	N2—C7—H7	125.4
C4—C3—C2	111.4 (3)	N1—C7—H7	125.4
C4—C3—H3A	109.3	C7—N1—C5	106.4 (3)
C2—C3—H3A	109.3	C7—N1—C2	126.5 (3)
C4—C3—H3B	109.3	C5—N1—C2	127.1 (3)
C2—C3—H3B	109.3	C7—N2—C6	109.9 (3)
H3A—C3—H3B	108.0	C7—N2—H2	125.1
O4—C4—O3	123.8 (3)	C6—N2—H2	125.1
O4—C4—C3	123.6 (3)	C4—O3—H3C	112.9

### Hydrogen-bond geometry (Å, °)

**Table:**

*D*—H···*A*	*D*—H	H···*A*	*D*···*A*	*D*—H···*A*
N2—H2···O2^i^	0.86	1.91	2.716 (4)	155
O3—H3C···O1^ii^	0.86	1.71	2.572 (3)	177

Symmetry codes: (i) *x*−1, *y*, *z*; (ii) *x*, *y*+1, *z*.
